# Determining the e-learning readiness of higher education students: A study during the COVID-19 pandemic

**DOI:** 10.1016/j.heliyon.2022.e11160

**Published:** 2022-10-19

**Authors:** Wagiran Wagiran, Suharjana Suharjana, Muhammad Nurtanto, Farid Mutohhari

**Affiliations:** a Department of Mechanical Engineering Education, Yogyakarta State University, Indonesia; b Department of Sports Science, Yogyakarta State University, Indonesia; c Department of Mechanical Engineering Education, Universitas Sultan Ageng Tirtayasa, Indonesia; d Department of Vocational and Technology Education, Yogyakarta State University, Indonesia

**Keywords:** Equipment capability, Higher education, Technological skills, User satisfaction, Motivation, e-learning readiness

## Abstract

The low readiness of university students to implement e-learning during the COVID-19 pandemic is a worrying issue. Lack of motivation and satisfaction in learning coupled with low technological skills are widely revealed as contributing factors. This study examines the role of technological skills, equipment capabilities, user satisfaction, and motivation on e-learning readiness. Furthermore, the study also examines the significance of the mediating role of motivation. The study adopted an ex-post-facto design involving 1052 students as participants. Data is collected from a questionnaire form integrated into the university's e-monev system. SEM-PLS is a data analysis tool with a confidence interval of 97.5%. After being analysed, technology skills, equipment capabilities, user satisfaction, and motivation are proven to play a role in e-learning readiness. Likewise, motivation also succeeded in proving its mediating role in this study. The study's results further clarify that efforts to improve e-learning readiness require digital technology capabilities, equipment capabilities, user satisfaction, and motivation, so vocational education must strengthen these aspects.

## 1 Introduction

Currently, digital technology in education is increasingly playing a comprehensive role in learning (Kashada et al., 2018; Sprenger and Schwaninger, 2021). The consequence is a change from the old learning mode to the e-learning mode (Ronzhina et al., 2021). Moreover, it is exacerbated by the pandemic conditions, which add important consequences to the use of e-learning, so it is not surprising that this mode is increasingly being applied (Choudhury and Pattnaik, 2020; Rodrigues et al., 2019). Although it has been widely defined, e-learning is a learning model that is systemized and based on the electronic web (Holmes and Gardner, 2006). This model integrates four important pillars in the educational process: learning media, ICT such as the internet, digital platforms, and video audio teleconferencing (Holmes et al., 2019). This learning model covers a well-structured learning process (Saripudin et al., 2020; Zare et al., 2016). A more efficient learning process is a major advantage so that access to important resources for learning is felt by both students and lecturers (Baumann-Birkbeck et al., 2015; Priatna et al., 2020). This efficiency is important in boosting learning effectiveness so that achievement is easier and faster (El-Sabagh, 2021). Various relevant studies agree that e-learning also has comprehensive features and allows for opportunities to apply an innovative, communicative, active, independent, reflective, and collaborative learning climate (Shah and Barkas, 2018; Wali and Popal, 2020). Likewise, teaching work has changed significantly because of the ease with which teachers can attend virtual classes anywhere without being present in a real room (Osman, 2020). In contrast to classical learning, this convenience has a direct impact on providing flexibility in time and place in teaching without reducing its quality (Lapitan et al., 2021).

At the beginning of the surge in cases of the COVID-19 Pandemic in 2020, the implementation of learning using e-learning resulted in a setback. Based on empirical research in developing countries, especially Indonesia, e-learning during the COVID-19 period resulted in two sides of the conflict: the negative impact as a setback and the positive impact as an increase. The main problems related to e-learning readiness include the lack of support capacity in the form of accessibility and facilities and the low ability to use devices for network access (Hamid et al., 2020). Besides that (Hamid et al., 2020) declared an astonishing finding that student involvement in e-learning is more than seventy percent less effective. This continues in other evidence that also identifies problems with the decline of e-learning, including fatigue in body organs (Octaberlina and Muslimin, 2020), psychological disorders, especially mental and motivational (Atmojo and Nugroho, 2020), and internet connection stability (Febrianto et al., 2020). Over the last two years, there has been a shift in positive areas, although not evenly distributed, namely the increasing use of technology and the internet, followed by digital literacy skills (Fathoni et al., 2021). This means that the readiness for the implementation of e-learning is increasing.

The most important thing to do is measure the readiness to implement e-learning before starting it (Gay, 2016; Widyanti et al., 2020). Several important studies agree that the results reveal that the basic reason for failure in implementing e-learning is the unpreparedness to implement e-learning (Coşkun et al., 2018; Widyanti et al., 2020). The implication is that measuring the level of student readiness must be done, starting from gap analysis to rearranging innovation to seek better integration of electronic learning (Alqahtani and Rajkhan, 2020; Nwagwu, 2020). Readiness in this context is also an assessment related to the overall availability of aspects in the realm of psychology, physical competence, and the availability of tools for the main learning needs (Holmes and Gardner, 2006). This is also confirmed by Mabrur et al. (2021), and Watkins et al. (2008) e-learning readiness is the level of physical readiness that refers to the infrastructure at the institution to implement the e-learning process. that e-learning readiness refers to basic capital (psychology and skills) in human resources and important infrastructure which are the main needs. All aspects incorporated in the readiness must at least be fulfilled (Oketch et al., 2014). However, the study from (Choudhury and Pattnaik, 2020; Terras and Ramsay, 2014) puts the main emphasis on preparing the provisions that students must have, considering that this is the most difficult thing to realize by the institution and the students themselves. The main aspect that becomes an important provision for students is none other than the ability of technology and equipment to support the implementation of e-learning (Al-araibi et al., 2019). According to Elkaseh et al. (2015), technological competence is necessary to support its comprehensive implementation. In addition (Coşkun et al., 2018; Nurtanto et al., 2021), indicate that the competence to use technology and its supporting equipment is a major requirement in implementing e-learning, so that they are able to experience comprehensive benefits from it. The benefits of comprehensive e-learning can be fulfilled when students and lecturers have the skills that make it easier for them to access and use internet-based digital technology.

In addition to the need for technological capabilities and supporting equipment, other aspects also need to review the readiness to carry out e-learning. Aspects of support from students that refer to their psychological conditions are very important in increasing e-learning readiness. Student satisfaction as a user of electronic lecture system services is an important aspect that must be provided by the institution (Pereira et al., 2015a, Pereira et al., 2015b). Student satisfaction as a user expresses students' feelings about the compatibility between expectations and reality received from lecture services. This suitability is very important to be given to students by the institution. Yilmaz (2017) states that spurring students to learn optimally during online learning is to monitor and evaluate their satisfaction. This is important as an effort to control the learning carried out by students so that their readiness for learning is high. To provide sufficient satisfaction during learning to students (Topal, 2016) identifies efforts that educational institutions can make, namely providing good facilities, infrastructure, and accessibility. Generally, online learning is an alternative method, but in the COVID-19 pandemic, it is the only effective and efficient way to consider the risk of transmission and the fatal impact. Thus, online learning becomes a primary need, and it is important to reveal the psychological condition of students. Thus, the shortcomings of online learning can be overcome.

Other psychological aspects were also identified by research (Yilmaz, 2017), namely learning motivation to strengthen students' willingness to online learning. Maldonado et al. (2011) define motivation as referring to the self-stimulation of learning activities. Furthermore, learning motivation was identified as being able to boost various learning needs, including needs in terms of learning readiness. Motivation is fundamental in stimulating activity for web-based and application-based electronic learning. This is relevant to the evidence from (Wang et al., 2021), which revealed that during distance learning during the COVID-19 pandemic, students decreased their learning readiness due to decreased motivation. Then (Fierro-Suero et al., 2020) interviewed several students, finding that they needed sufficient motivation to navigate electronic learning. However, learning motivation has a different direction, namely increasing motivation and decreasing motivation due to stress conditions or loads that exceed normal limits. Finally, both orientations have the potential to focus on learning during the COVID-19 pandemic. So, students' motivation in e-learning is one of the important considerations to be involved.

The focus of this study is to link facilities, digital literacy, and psychological aspects of students as a form of their readiness to use e-learning during COVID-19. In simple terms, measuring technology capabilities, availability of supporting facilities, user satisfaction, and student motivation while involved in e-learning learning. Various empirical studies have conceptualized e-learning in a pandemic period from the aspect of success and failure (see Table 1). In developing countries, the success and failure of e-learning are determined from the aspect of accessibility and facilitation related to technology with low awareness. Furthermore, student motivation factors affect the success and failure of e-learning. Finally, the e-learning service satisfaction factor occupies the third largest factor compared to other factors. Satisfaction was identified as a growth factor for students' motivation in using e-learning. These results motivate researchers to research because the main reason for the success or failure of e-learning comes from e-learning readiness which is also caused by aspects of technology, service satisfaction, and motivation. This study aims to analyze the effect of technological skills, equipment capabilities, user satisfaction, and motivation on the e-learning readiness of college students. In addition, the researcher also examines the role of user satisfaction and motivation as a mediator.

**Table 1:** Conceptualization of the use of e-learning in higher education in the COVID-19 era.

E-learning Comparison	Aspects of E-learning	References
E-learning success	Technological roles	(Adams et al., 2022; Alqahtani and Rajkhan, 2020; Astuti et al., 2022; Mabrur et al., 2021; Mutohhari et al., 2021; Nasution, 2021; Nwagwu, 2020; Putro et al., 2021)
	Learning environment	(Adams et al., 2022)
	Learning styles	(Yavuzalp and Bahcivan, 2021)
	Learning achievement	(Nwagwu, 2020; Yavuzalp and Bahcivan, 2021)
	Equipment capabilities	(Adams et al., 2022; Aldhahi et al., 2022; Mabrur et al., 2021; Putro et al., 2021)
	Family support	(Nwagwu, 2020)
	Peer support	(Nwagwu, 2020)
	Student satisfaction	(Yavuzalp and Bahcivan, 2021)
	Completeness of infrastructure	(Adams et al., 2022; Mabrur et al., 2021; Nwagwu, 2020)
	Flexible and effective learning	(Gurban and Almogren, 2022; Mukhtar et al., 2020)
	Easy accessibility	(Gurban and Almogren, 2022; Mukhtar et al., 2020)
E-learning failures	Lack of technology skills	(Adams et al., 2022; Aldhahi et al., 2022; Mabrur et al., 2021; Putro et al., 2021)
	Lack of digital literacies	(Adams et al., 2022; Alqahtani and Rajkhan, 2020)
	Lack of Teaching quality	(Yavuzalp and Bahcivan, 2021)
	Lack of interaction	(Yavuzalp and Bahcivan, 2021)
	Lack of resources	(Adams et al., 2022; Mabrur et al., 2021)
	Lack of self-efficacy	(Adams et al., 2022)

## 2 Theoretical background

### 2.1 Equipment capability and technological skills in user satisfaction

Student satisfaction is an important investment for the future of higher education that can improve quality and assist in achieving its goals (Fatani, 2020). High satisfaction is one indicator of fulfilling most aspects of students' needs in learning. Student satisfaction can also be used as a reference to direct management toward meeting student needs in learning (Sholikah and Harsono, 2021). Satisfaction indirectly affects learning and teaching activities, so the high and low outcomes are mostly associated with these factors (Nwagwu, 2020; Yilmaz, 2017). Student satisfaction is most influenced by the quality of university services (Annamdevula and Bellamkonda, 2016). Various service aspects are considered important and related to increasing student satisfaction as service users. The aspect of self-development is one of the important services revealed by various previous studies in increasing user satisfaction (Chandra et al., 2018; de Jager and Gbadamosi, 2013; Narindro et al., 2020). Self-development refers to the development of skills considered important to meet the needs of students in supporting the learning process (Chandra et al., 2018; Htang, 2021).

In technological developments in the 21^st^ century and exacerbated by the COVID-19 pandemic, which has shifted the mode of learning towards e-learning, it is an important issue that the development of skills-therapeutic skills are needed to support e-learning learning, so that it ends up also increasing student satisfaction (Alqurashi, 2019; Fawaid et al., 2022; Puška et al., 2021). Thus, higher education is very important to develop skills that play an important role in supporting e-learning. Skills in using technology and supporting equipment are the most important aspects (Chitkushev et al., 2014). Technological skills refer to the basic level to the highest level and depth of digital technology (Arifin et al., 2020; Astuti et al., 2022; Mutohhari et al., 2021; Sutiman et al., 2022). Pavlova (2009) classifies five important skills in using digital technology, namely awareness, literacy, capability, and creativity, and they are critical in using digital technology. The higher the level of digital technology skills, the more comprehensive the ability of students to use e-learning services so that the estuary will affect student satisfaction as users of these services (Shehzadi et al., 2021; Sholikah and Harsono, 2021). Coşkun et al. (2018) describe the process of forming student satisfaction which originally started with the fulfillment of aspects that support the achievement of their needs, including the development of technological skills in students.

Developing digital technology skills in students aligns with the skills needed for learning in the 21^st^ century (Ronzhina et al., 2021). Several studies agree that learning dominated by distance learning, such as e-learning requires high digital technology skills (Astuti et al., 2022; Gafurov et al., 2020; Yureva et al., 2020). Students must know and master basic skills and be in-depth, creative, and critical in e-learning. This is because various media and digital learning resources require a comprehensive study assisted by digital technology skills to obtain high effectiveness and efficiency. In addition, digital technology skills also provide understanding in mastering the supporting tools for running e-learning today (Almaiah et al., 2020; Elkaseh et al., 2015). Pereira et al., 2015a, Pereira et al., 2015b revealed that skills in using supporting equipment, such as computers, the internet, software, and applications, need digital technology skills. This then provides the main capital for students undergoing electronic learning to meet learning needs, and the estuary also increases their satisfaction.

H1 There is a significant effect of technological skills on the equipment capability

H2 There is a significant effect of technology skills on the satisfaction of users.

H3 There is a significant effect of equipment capability on users' satisfaction.

### 2.2 Technological skills, equipment capability, and user satisfaction in motivation

In any learning mode, student motivation plays an important role in learning success (Malinauskas and Pozeriene, 2020; Wu et al., 2021). Moreover, in the era of distance learning using e-learning, students' learning motivation is seen as a crucial factor that forms the basis for such learning (Almaiah et al., 2020; Ferrer et al., 2022). Motivation is a student's psychological process that stimulates the formation of an urge to do an activity (Hoffman, 2015). Motivation is seen as forming the spirit and mentality of students in learning. High or low motivational support from students will fluctuate in open-mindedness and actions to absorb knowledge and skills in learning (Alemayehu and Chen, 2021; Rafiola et al., 2020; Yang et al., 2018). Several previous studies have revealed various crucial problems in online learning: students' lack of motivation to learn. They admit that they lack the motivation to learn due to the shift in learning modes without strong capital, which is their need to deal with the shift (Hamid et al., 2022; Rasmitadila et al., 2020). Elkaseh et al. (2015) revealed the results of their review of various research report articles, which simultaneously stated that the technological aspect, including digital technology skills and the ability to use supporting equipment, is the highest aspect that affects e-learning learning motivation.

On the other hand (Al-araibi et al., 2019), also identified the lack of formation of the technological aspects of the students behind the low motivation of students to learn so that in undergoing e-learning, they were not ready. This indicates that students must have high acceptance in mastering digital technology and its supporting equipment. As previously described (Astuti et al., 2022; Pavlova, 2009), agreed on five important digital technology skills that must be mastered starting awareness, literacy, capability, creativity, and critical use of digital technology, so this is a consideration that students must have a complete acceptance of digital technology to increase motivation in running e-learning. Awareness supported by literacy and capability will equip students to use digital technology appropriately according to the procedures to support e-learning (Falloon, 2020). Nevertheless, the need for creativity and criticality in using digital technology aims to provide better efficiency and effectiveness in e-learning (Fletcher et al., 2020; Hoffmann et al., 2016). In addition, the level of creativity and criticality of students will affect their breadth and depth of learning through e-learning, which is also in line with the formation of student learning motivation (Elkaseh et al., 2015). Their review reported that the need for creativity and depth in using digital technology for learning will provide a broader way of thinking and have more comprehensive benefits (van Laar et al., 2017). In addition, many digital learning resources in the e-learning process require students' filtering and evaluation skills to get credible learning resources according to what they are studying (Trilling and Fadel, 2012). This is what they will get if the acceptance of the technology reaches the creativity and critical level (Haryanto et al., 2021; Nurtanto et al., 2020). Then, Hava (2021) explains the impact of increasing student learning motivation after they have a high acceptance of digital technology abilities. Thus, the acceptance of digital technology, which also includes the ability to operate supporting equipment, is very important to students to increase motivation during learning, especially in e-learning.

H4 There is a significant effect of technology skills on motivation.

H5 There is a significant effect of equipment capability on the motivation

Apart from strengthening the technological aspect, student motivation in learning through e-learning is also motivated by providing student satisfaction as users of e-learning services (Yılmaz, 2022). Student satisfaction as a user is defined as feeling happy for students regarding the suitability between expectations and reality received from lecture services (Htang, 2021). This suitability is very important to provide, considering that students will feel satisfied if what they hope to support learning is fulfilled, especially in conditions of e-learning during a pandemic (Chitkushev et al., 2014; Pereira et al., 2015a, Pereira et al., 2015b). El-Seoud et al. (2014) prove that learning support services provide a positive signal as a driver for students to implement e-learning optimally. In providing optimal satisfaction of electronic lecture services to students (Sholikah and Harsono, 2021; Topal, 2016), identify educational institutions' efforts, namely providing good facilities, infrastructure, and accessibility. In addition, student satisfaction will also be felt if educational institutions can become good problem-solvers in overcoming online learning problems for students (Chandra et al., 2018). Fast and quality responses and feedback must also be principles and actions that must be taken (Dziuban et al., 2019). The existence of student satisfaction that departs from these aspects will certainly encourage students to study diligently using the e-learning system.

H6 There is a significant effect of user satisfaction on motivation.

### 2.3 Technological skills, equipment capability, user satisfaction, and motivation in E-learning readiness

In the last decade, aspects of digital technology have become the background for the high and low readiness of students in universities in carrying out e-learning. The digital technology aspect, defined as digital technology competence, takes on an important role, including operational skills and supporting equipment (Al-araibi et al., 2019). More than a year into the COVID-19 pandemic, research trends and literature reviews have identified a consistent problem, namely the unpreparedness of learning caused by the lack of basic supplies related to technological skills to accommodate e-learning (Bhuasiri et al., 2012; Sugandini et al., 2022; Valverde et al., 2020). The lack of this refers to low awareness and lack of operational skills in learning support technology and is exacerbated by the crisis of attitudes and ethics in digital technology. In the same vein, the identification and observations of other researchers agree that referring to the absence of technological competence is an antecedent factor of student unpreparedness in learning, thus requiring the development of these competencies (Nguyen et al., 2019). Elstad and Christophersen (2017) claim that basic and comprehensive digital technology competency-based training is an absolute must to foster experience and readiness in running e-learning. Thus, these descriptions provide high confidence to researchers to draw tentative conjectures that have the potential to be proven in the results of research data analysis.

H7 There is a significant effect of technology skills on e-learning readiness

H8 There is a significant effect of equipment capability on e-learning readiness.

Developing competence in students in line with learning needs is very important. However, there are also important aspects that must also be given to balancing the development of competencies. The aspect of student satisfaction as service users must be a guarantee and commitment of higher education institutions to increase their students' readiness to do online learning (Alqurashi, 2019). As previously defined, student satisfaction is directly related to psychological conditions, with feelings of happiness as the fruit of the conformity of expectations with the reality given to them to support learning (Htang, 2021). This suitability is very important to provide, considering that students will feel satisfied if what they hope to support learning is fulfilled, especially in conditions of e-learning during a pandemic (Chitkushev et al., 2014). Yilmaz (2017) revealed that student satisfaction with e-learning system services would foster positive attitudes and encourage students to learn maximally using the system to build motivation. Then, in addition to guaranteeing student satisfaction, universities must also strengthen student motivation in learning to use e-learning (Fierro-Suero et al., 2019). A strong internal drive plays a basic function in equipping students with a willingness to learn, so it can allegedly boost e-learning readiness in students (El-Seoud et al., 2014). Terras and Ramsay (2014) define the motivation of e-learning in students as the psychological drive that spurs students to be active in the electronic learning process. Motivation is needed to give students enthusiasm and mentality to learn to use computer media, the internet, and related software. Readiness in learning will increase when students have a good background of will and enthusiasm. Besides that, his mentality is also optimally boosted (Truzoli et al., 2021; Yilmaz, 2017). Based on these descriptions, the researcher draws tentative conjectures that have the potential to be proven in the results of the research data analysis.

H9 There is a significant effect of user satisfaction on e-learning readiness.

H10 There is a significant effect of motivation on e-learning readiness.

Although it has a significant effect on e-learning readiness, the technological aspect that includes digital technology skills and supporting equipment requires a motivational aspect to be the mediator, as well as in the aspect of user satisfaction. The intervention variable used is that the process of forming e-learning readiness is not directly influenced by aspects of technology and job satisfaction alone. Technological aspects and job satisfaction can form motivation first before forming e-learning readiness in students. Research from (de Barba et al., 2016; Pan, 2020; Wang et al., 2021) reports that motivation plays a significant role in mediating the indirect effect of digital technology capabilities and supporting equipment on e-learning readiness. They concluded that technological competence indirectly affects e-learning readiness because learning motivation is performed. In line with this (Bailey et al., 2021; Goulimaris, 2015), his research also tested the significance of the mediating role of learning motivation in mediating the effect of job satisfaction on distance learning readiness. They concluded that motivation is a fundamental aspect that must be possessed when carrying out online learning. Meanwhile, job satisfaction is the strongest factor that provides a strong impetus for the readiness to use e-learning.

H11 There is a significant effect of digital technology capabilities on e-learning readiness through the mediation of motivation.

H12 There is a significant effect of supporting equipment on e-learning readiness through the mediation of motivation.

H13 There is a significant effect of user satisfaction ability on e-learning readiness through the mediation of motivation.

**Figure 1:**
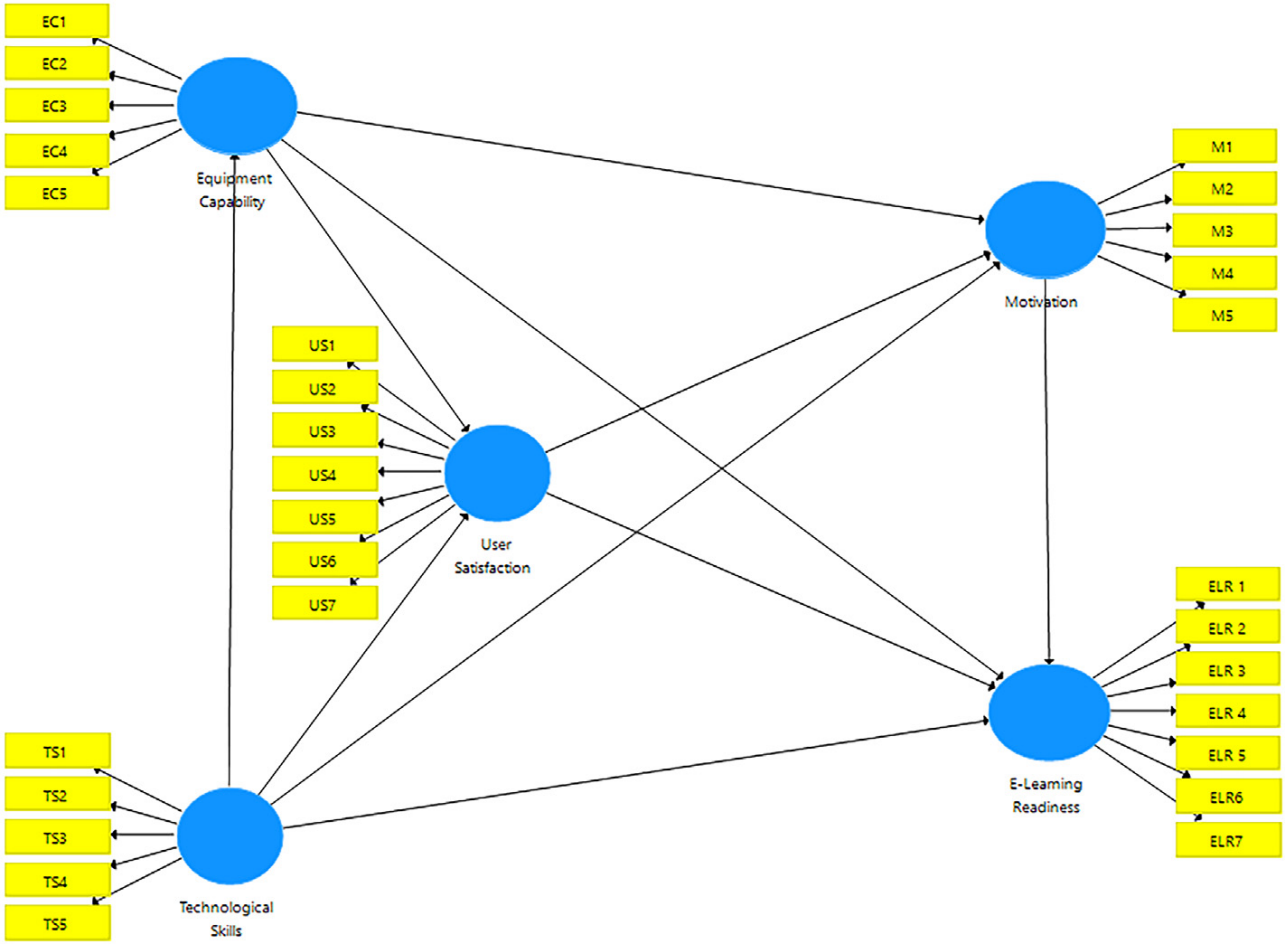
Conceptual framework of e-learning readiness.

The direct relationship between variables and the mediated relationship is presented in Figure 1 as an interpretation of the hypothesis described above.

## 3 Method

### 3.1 Research design

Considering the data and the research area, we adopted an ex-post facto research method, the design of which was developed by Cohen et al. (2011). This is a cross-sectional study in which data were collected through a questionnaire designed with structured questions. Following the conceptual framework and existing theoretical studies, mediation's direct influence and role are measured based on the actual data. The analyzed data reflects their respective roles in improving college students' e-learning readiness. We ensure the direction of the research is in line with the duration of e-learning implementation based on certain time criteria (during the online learning policy, April 2020 to July 2021) so that participants who fill out the questionnaire have sufficient experience in this regard.

### 3.2 Participants

The respondents involved in this study were undergraduate students in Yogyakarta, Indonesia. The purposive sampling technique was used to distribute questionnaires (Tongco, 2007) online, considering that data collection was still in the COVID-19 the last period in 2021. Inclusion criteria were set e-learning students from various semesters were grouped, only selected in three fields, namely Tourism, IT, and Technology and Engineering, which are intended to facilitate decision-making on the implementation of e-learning for Engineering students. Other criteria also consider the level of intensity of use in a week. Exclusion criteria were applied to reduce responses that did not meet the initial criteria. After the data was collected, we ensured the validity of the participants in filling out the previous questionnaire in e-monev. After considering, at least sixty-eight entries have low validity, including those that have relatively the same answers on all items, considering that they are only completed in less than 30 s. In the end, we included a final sample of 1052 with a 95% representativeness of the response. Detailed background data of our participants are presented in Table 2.

**Table 2:** Background of participants.

Dimensions	Category	Frequency	Percentage
Gender	Male	594	56.46%
	Female	458	43.54%
Study period	1–4 semesters	448	42.59%
	5–8 semesters	394	37.45%
	9–12 semesters	162	15.40%
	13–14 semesters	48	4.56%
Expertise	Tourism	323	30.70%
	IT	348	33.08%
	Technology and Engineering	381	36.22%
E-learning intensity	8–12 h a week	139	13.21%
	13–17 h a week	166	15.78%
	18–22 h a week	319	30.22%
	23–27 h a week	528	50.19%

### 3.3 Data collections and measurements

Instruments are generated from the relevant review literature, then discussed together by involving the entire research team and a team of experts to get input on the feasibility of the content. Furthermore, the questionnaire was registered with the research ethics commission at the Institute for Research and Community Service (LPPM) at Yogyakarta State University to obtain ethical approval. Then, the questionnaire is compiled in an online monitoring and evaluation (e-monev) system and is ready to be distributed to students at each university.

Data is collected through each university's monitoring and evaluation system (e-monev). Although we have expanded the sample from various semesters, the period from October to December 2021 was chosen considering that students have re-adapted to learning at least 8 meetings. Researchers with the e-monev admin coordinated to obtain data from complete sample data. To ensure completeness, we also confirmed that the system is related to access to grades in the academic information system, so students must fill out a questionnaire before accessing it. Then don't forget, briefing students is also carried out to provide understanding related to technical filling and regulations so that data is obtained as rationally as possible. The data is in the form of raw data from all participants recorded in the e-monev system. We adopted a measurement scale with a five-point Likert Scale ranging from “strongly disagree” to “strongly agree” as appropriate consideration. The survey items have ascertained the level of validity and reliability by adopting various relevant research, and we have retested them.

3.3.1. The technological skills instrument is based on the development of indicators by Astuti et al. (2022); Pavlova (2009), concerning five levels of the technology taxonomy, namely: “Awareness of the development of digital technology has been embedded in me,” “I have a comprehensive understanding of digital technology. ”, “I *can operate digital devices”, “The creativity of digital technology-based learning has been awakened in me”, and “I can choose the right technology for distance learning.*”
3.3.2. Equipment capability was also constructed with five question items which were developed based on the opinions by Elstad and Christophersen (2017), which consisted: “*I have appropriate digital technology devices to support learning*”, “*I have competence in using digital technology devices*”, “*I have good internet access to support digital platform operations*”, “*I have financing for the availability of ideal access devices*”, and “I have secondary devices to support the operation of an application, such as headphones, speakers, microphones, and others”.
3.3.3. User satisfaction developed based on this opinion by Yilmaz (2017) consists of seven statement items which contain: *“The electronic web-based lecture process guarantees my satisfaction", "I feel that student problem-*solving services are well managed”, “The ease of taking care of administration gives satisfaction to students. I can feel the facilities provided by the institution to boost the learning process”, “Relevant lecturers provide intensive services in various problems that I experience”, “The friendliness and courtesy of the officers in the academic environment give me satisfaction”, and “I satisfied with the speed and accuracy of the officers in providing feedback”.
3.3.4. Motivation is measured based on five measurements that Yilmaz (2017) has previously developed including: “I have good concentration during distance learning”, “Challenges and new opportunities in online learning encourage me to study harder”, “Problems in learning-based learning”*,* “*The electronic web gives me the enthusiasm to solve it*”, “*The impetus has always been with me as a passion in completing tasks*”, and “*I have a good urge to expand access to learning resources using internet-based digital devices*”.
3.3.5. Finally, e-learning readiness adopts a readiness scale by Adams et al. (2022) and Yilmaz (2017), which consists of seven statement items, including: “I have self-efficacy to be able to use information and communication technology (ICT)”, “Self-efficacy in managing distance learning has been built", "My self-confidence has been built through the use of internet and ICT media”, “I already have the expertise in accessing and managing electronic learning systems”, “A strong urge has been awakened in me to adopt distance learning mode”, and “*The effectiveness of learning outcomes is a target that I am ready to achieve*”.

### 3.4 Data analysis

Structural Equation Modeling (SEM) analysis was used to test the hypothesis of a direct influence between variables and the role of mediation with a certain level of confidence (97.5%). The analysis was carried out twice with different methods using the help of the SmartPLS software version 3.0. First, the analysis results are determined by obtaining standardized estimation coefficients and probability values for each path. It refers to the direct effect of each exogenous variable on the endogenous variable as defined. Then, finally, the results of the mediation role test results are determined by obtaining bootstrap analysis, considering that the method is the most rational and can obtain the minimum error limit (Preacher and Hayes, 2008).

## 4 Result

### 4.1 Instrument validity and reliability

Data were collected based on filling out the instrument in the e-monev system, but the instrument was tested for validity and reliability before being analyzed further. Validity and reliability were tested constructively and analyzed using Confirmatory Factor Analysis and Cronbach Alpha methods and put the minimum standard number on the outer loading coefficient and reliability index. The threshold of validity is set at 0.700 (OL ≥ 0.700), and the validity is considered to be fulfilled if the value is at least 0.800 (α ≥ 0.800) (Johnson and Wichern, 2007). The analysis resulted in good validity and reliability on all instrument items. This means that the data generated from filling in all items met the eligibility for further analysis (Preacher and Hayes, 2008). Table 3 details the validity and reliability tests results based on the outer loading value and the alpha value. Likewise, the reliability test detailed in Table 4 obtained a reliability index with very high criteria on all instruments (Reid, 2014). Both provide certainty that the instrument has good feasibility for further data collected through all items can be analyzed further.

**Table 3:** Instruments validities.

Aspect	Indicator	Outer Weight	Outer Loading	Decision
Technological skills	TS1	0.230	0.864	Good
	TS2	0.218	0.883	Good
	TS3	0.234	0.885	Good
	TS4	0.252	0.826	Good
	TS5	0.781	0.870	Good
Equipment capability	EC1	0.273	0.770	Good
	EC2	0.231	0.716	Good
	EC3	0.261	0.815	Good
	EC4	0.309	0.846	Good
	EC5	0.215	0.702	Good
User satisfaction	US1	0.168	0.778	Good
	US2	0.190	0.812	Good
	US3	0.176	0.850	Good
	US4	0.186	0.813	Good
	US5	0.177	0.843	Good
	US6	0.218	0.795	Good
	US7	0.321	0.742	Good
Motivation	M1	0.238	0.783	Good
	M2	0.248	0.829	Good
	M3	0.211	0.739	Good
	M4	0.262	0.862	Good
	M5	0.276	0.817	Good
E-learning readiness	ELR1	0.175	0.887	Good
	ELR2	0.159	0.843	Good
	ELR3	0.178	0.900	Good
	ELR4	0.167	0.885	Good
	ELR5	0.146	0.839	Good
	ELR6	0.160	0.867	Good
	ELR7	0.162	0.875	Good

**Table 4:** Instruments reliabilities.

Aspect	Alpha	rho_A	CR	AVE	Decision
Technological skills	0.917	0.949	0.957	0.759	High
Equipment capability	0.829	0.842	0.880	0.595	High
User satisfaction	0.909	0.872	0.903	0.652	High
Motivation	0.866	0.918	0.937	0.750	High
E-learning readiness	0.947	0.911	0.928	0.649	High

### 4.2 Model fit test

**Figure 2:**
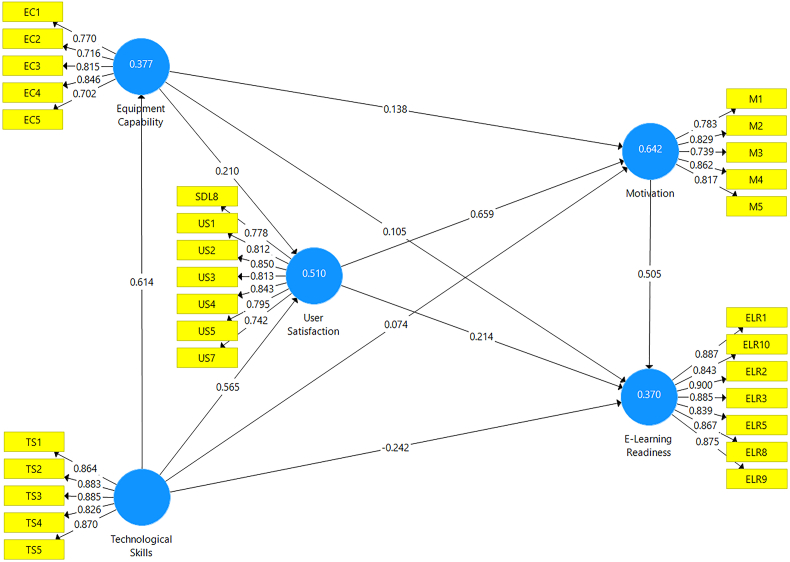
Structural model analysis results.

The model suitability test justifies the level of conformity of the structural model used so that the model can explain the structural coefficients of the relationship between variables. The overall fit index of the research model is presented (as the baseline model), as shown in Table 5. As presented, all the overall fit index of the baseline model performed well. The expected small chi-squared value is well realized. The high probability value (p-value 0.050) explains that there is no difference between the model being tested and the data, so the model is declared capable of predicting the value of its observations (Streiner, 2005). GFI, AGFI, CFI, and NFI all performed above the threshold value (≥0.90). SRMR < 0.05 and RMSEA < 0.08, so it is concluded that the model has high suitability and structural model analysis can be carried out (Westland, 2019). The structural analysis model used is presented in Figure 2.

**Table 5:** Model fit test result.

The goodness of fit aspect	Result	Evaluation
Chi-square	13.501	Small (fit)
Probability	0.592	>0.50 (fit)
GFI	0.905	≥0.90 (fit)
AGFI	0.931	≥0.90 (fit)
CFI	0.912	≥0.90 (fit)
TLI	0.903	≥0.90 (fit)
SRMR	0.028	<0.05 (fit)
RMSEA	0.051	<0.08 (fit)

### 4.3 Direct effect test

Hypothesis testing 1–10 is seen based on the standardized value of the path coefficient and probability. Testing the confidence interval obtained a very good percentage of 97.5% and an error limit of 2.5%. The results of this analysis comprehensively present the direct influence of technology skills on equipment capabilities, technological skills and equipment capabilities on user satisfaction, technology skills, equipment capabilities, and user satisfaction on motivation, as well as technology skills, equipment capabilities, and user satisfaction on motivation. Technology skills, equipment capabilities, user satisfaction, and motivation towards e-learning readiness. The test was carried out using the entire sample, and the samples were grouped on each participant's background. Testing on the sample on the background variation is carried out to test whether the hypothesis can be tested on all respondents with various characteristics, as shown or not. Table 6 details all standardized path coefficients with the overall sample, while Table 7 uses samples based on each dimension of the participants' background.

**Table 6:** Path analysis test result.

Path	Standardized			
	Estimate	t value	SE	p
Equipment capability → e-learning readiness	0.105	2.841	−0.001	0.005
Equipment capability → motivation	0.138	4.519	0.000	0.000
Equipment capability → user satisfaction	0.210	6.917	0.000	0.000
Motivation → e-learning readiness	0.505	10.502	0.002	0.000
Technological skills → e-learning readiness	0.242	5.595	−0.001	0.000
Technological skills → equipment capability	0.614	26.596	0.000	0.000
Technological skills → motivation	0.074	2.182	0.000	0.029
Technological skills → user satisfaction	0.565	19.876	0.000	0.000
User satisfaction → e-learning readiness	0.214	4.257	0.000	0.000
User satisfaction → motivation	0.659	21.484	0.000	0.000

P < 0.05.

**Table 7:** Path analysis results according to participant background.

H	p-Value												
	Gender		Age range (years)				Expertise			E-learning intensity (h)			
	M	F	18–19	20–21	22–23	24–25	TE	ICT	T	8–12	13–17	18–22	23–27
1	0.001	0.002	0.000	0.000	0.001	0.000	0.000	0.000	0.000	0.001	0.000	0.000	0.000
2	0.000	0.000	0.006	0.000	0.000	0.000	0.000	0.002	0.000	0.000	0.000	0.000	0.000
3	0.001	0.000	0.000	0.000	0.000	0.000	0.000	0.000	0.000	0.000	0.000	0.000	0.000
4	0.021	0.009	0.012	0.040	0.034	0.041	0.006	0.008	0.000	0.000	0.000	0.041	0.032
5	0.000	0.000	0.000	0.000	0.000	0.000	0.001	0.000	0.000	0.001	0.002	0.000	0.004
6	0.001	0.000	0.000	0.000	0.000	0.000	0.000	0.000	0.003	0.000	0.000	0.000	0.000
7	0.006	0.002	0.000	0.000	0.008	0.011	0.000	0.001	0.000	0.000	0.000	0.000	0.007
8	0.000	0.000	0.000	0.000	0.000	0.000	0.000	0.000	0.000	0.000	0.000	0.000	0.000
9	0.000	0.000	0.000	0.000	0.000	0.000	0.000	0.000	0.000	0.000	0.000	0.000	0.000
10	0.000	0.000	0.000	0.000	0.000	0.000	0.000	0.000	0.000	0.000	0.000	0.000	0.000

Technological skills affect the equipment's ability with an estimated standardized path coefficient of 0.614 and a probability of 0.000. Therefore, H1 is supported. Then technology skills also affect user satisfaction with the estimated standardized path coefficient value of 0.565 and probability of 0.000, thus supporting H2. The equipment's ability affects user satisfaction with the estimated standardized path coefficient value of 0.210 and probability of 0.000, thus supporting H3. Technological skills affect motivation with an estimated standardized path coefficient of 0.074 and a probability of 0.029, thus supporting H4. The estimated standardized value of the path coefficient is 0.138, and the probability is 0.000, shown on the line of the equipment capability on the motivation to support H5. Then user satisfaction increases motivation with an estimated standardized path coefficient of 0.659 and a probability of 0.000, thus supporting H6. Technological skills affect e-learning readiness with an estimated standardized path coefficient of 0.242 and a probability of 0.000, so H7 is supported. Thus the estimated value of the standardized path coefficient of equipment capability toward e-learning readiness is 0.105, and the probability is 0.005, supporting H8. Then user satisfaction affects e-learning readiness with an estimated standardized path coefficient of 0.214 and a probability of 0.000. Therefore, H9 is supported. Likewise, motivation affects e-learning readiness with an estimated standardized path coefficient of 0.505 and a probability of 0.000, thus supporting H10.

### 4.4 Mediating roles test of motivation

Table 8 shows the role of motivation in mediating technology skills, equipment capabilities, and user satisfaction in influencing students’ e-learning readiness in higher education. The confidence interval obtained in this bootstrap method is 97.5%. The test results of the mediating role of motivation on the path between technology skills and e-learning readiness resulted in a standardized bootstrapping estimation value of 0.037 and a probability of 0.034. Based on these results, it is proven that motivation can mediate well on this pathway, so H11 is supported. Furthermore, the same results are also evident when motivation mediates the path of equipment capability and e-learning readiness with a standardized bootstrapping estimation value of 0.070 and a probability of 0.000, for that H12 is supported. The same results are also shown from the results obtained that there is an indirect effect of user satisfaction on e-learning readiness through mediation from motivation with a standardized bootstrapping estimation value of 0.334 and a probability of 0.000. Therefore H13 is also supported. Finally, Table 9 shows the effect of the mediating role of motivation based on each dimension in the participants’ background. Overall, it is explained that participants get a significant indirect effect on all dimensions in the background.

**Table 8:** Mediating roles of motivation.

Mediation pathway	Standardized coefficient with Bootstrapping (97,5 % CI)					
	Direct effect		Indirect effect		Total effect	
	Estimate	Sig	Estimate	Sig	Estimate	Sig
Technological skills → motivation	0.074	0.029	–	–	0.074	0.029
Technological skills → e-learning readiness	0.242	0.000	0.037	0.034	0.279	0.000
Equipment capability → motivation	0.138	0.000	–	–	0.138	0.000
Equipment capability → e-learning readiness	0.105	0.005	0.070	0.000	0.175	0.000
User satisfaction → motivation	0.659	0.000	–	–	0.659	0.000
User satisfaction → e-learning readiness	0.214	0.000	0.334	0.000	0.548	0.000

**Table 9:** Mediating roles of motivation according to participant background.

Category	p-Value Standardized with Bootstrapping (95% CI)								
	Direct Effect			Indirect Effect			Total Effect		
	H7	H8	H9	H11	H12	H13	H7,11	H8,12	H9,13
M	0.006	0.000	0.000	0.038	0.004	0.000	0.000	0.000	0.000
F	0.002	0.000	0.000	0.031	0.000	0.000	0.000	0.000	0.000
18–19 y	0.000	0.000	0.000	0.038	0.001	0.000	0.000	0.000	0.000
20–21 y	0.000	0.000	0.000	0.04	0.000	0.000	0.000	0.000	0.000
22–23 y	0.008	0.000	0.000	0.037	0.000	0.000	0.000	0.000	0.000
24–25 y	0.011	0.000	0.000	0.034	0.002	0.000	0.000	0.000	0.000
T	0.000	0.000	0.000	0.028	0.003	0.000	0.000	0.000	0.000
ICT	0.001	0.000	0.000	0.021	0.000	0.000	0.000	0.000	0.000
TE	0.000	0.000	0.000	0.042	0.000	0.000	0.000	0.000	0.000
8–12 h	0.000	0.000	0.000	0.039	0.000	0.000	0.000	0.000	0.000
13–17 h	0.000	0.000	0.000	0.035	0.000	0.000	0.000	0.000	0.000
18–22 h	0.000	0.000	0.000	0.014	0.000	0.000	0.000	0.000	0.000
23–27 h	0.007	0.000	0.000	0.048	0.001	0.000	0.000	0.000	0.000

## 5 Discussion

The COVID-19 pandemic has lasted for a long time and has developed how college students think about implementing e-learning. Strategies for adaptation and intensity of use have increased quite dramatically (Adams et al., 2022; Çevik and Bakioğlu, 2022). In this research sample, most students (50.19%) have intensively studied e-learning every week, with an intensity of 23–27 h per week. These data indicate that students have adopted electronic learning. However, there are still (13.21%) who only use e-learning in the range of 8–12 h per week, making it less intensive. There are also students (19.96%) who fall within the graduation range above the standard (8 semesters), indicating that they are experiencing problems with graduation. The sample, in this case, is very heterogeneous, so it produces information that is also varied but not significant. The study findings reveal a significant influence between technology skills, equipment capability, user satisfaction, and student motivation on e-learning readiness. The next finding is that motivation becomes a significant mediator in technology skills, equipment capability, and user satisfaction with e-learning readiness during the COVID-19 pandemic.

Technological skills directly have a significant effect on equipment capability and user satisfaction. In line with that, the capability of the equipment also has a significant effect on user satisfaction. This evidence indicates the effectiveness of the maturation of digital technology skills in students to increase their ability to use e-learning support equipment so that the estuary also increases their satisfaction as service users. In carrying out e-learning, technology skills are needed to support the accessibility and use of digital technology equipment. In the same vein, relevant research has previously proven that digital technology skills are a major requirement in electronic-based learning, considering that there has also been an increase in the use of required supporting equipment (Díaz et al., 2019). Likewise, research in several countries confirms that satisfaction plays a role as an aspect that becomes a benchmark for increasing technological skills and equipment capabilities so that the maturation of technology skills and supporting equipment is in line with increasing student satisfaction as service users (Holmes et al., 2019; Ouajdouni et al., 2021; Yawson and Yamoah, 2020).

Taken together, technology skills, equipment capabilities, and user satisfaction have a significant effect on motivation. These results indicate that students need the development of e-learning support skills to stimulate motivation. In the same context, an intensive drive in learning grows because students are satisfied with existing services, including technology and equipment skills development services. These results prove, as well as confirm previous research, that skills in using digital technology slowly stimulate the growth of motivation during distance learning (Beardsley et al., 2021). The results of this study are also supported by research from (Alemayehu and Chen, 2021) which emphasizes the importance of strengthening accessibility skills of web-based digital platforms using supporting devices. In the same context, several researchers reaffirm that efforts to mature competence in students can stimulate the emergence of motivation, considering that their satisfaction is indirectly guaranteed (Fryer and Bovee, 2016; Juan-Lázaro and Area-Moreira, 2021).

The significant effect of technology skills, equipment capabilities, user satisfaction, and motivation on e-learning readiness is also revealed in this study. The significance of this influence is motivated by the main need for aspects of the use of e-learning. Readiness to adopt several electronic learning tools certainly requires digital-based skills and supporting equipment (Pavlova, 2009). In the same vein, researchers from several countries have shown the importance of digital web-based training and mentoring for navigating distance learning (Aliway and Safie, 2018; Valverde-Berrocoso et al., 2020). Furthermore, the attention given by previous researchers also points to the importance of ensuring the convenience of lecture services during online learning that is oriented to student satisfaction (Holmes et al., 2019; Vate-U-Lan, 2020). This is intended to provide student satisfaction while learning during the COVID-19 pandemic. This satisfaction refers to providing training in using e-learning comprehensively, both digital technology training and supporting equipment. Thus, student satisfaction from these aspects will ensure the convenience and comfort of students, according to the context studied previously (Fierro-Suero et al., 2019).

## 6 Conclusion and limitations

This study has proven the effect of variable technology skills, equipment capabilities, user satisfaction, and motivation on the e-learning readiness of college students. Based on the analysis of the whole sample and the sample with their respective characteristics, they have revealed the importance of the variables studied to reveal the factors that affect the readiness for e-learning in college students. In line with that, it is proven that motivation can mediate the effect of technological skills, equipment capabilities, and user satisfaction on e-learning readiness. The findings in this study indicate the importance of strengthening digital technology skills and supporting equipment before using e-learning. On the other hand, student satisfaction as service users during online learning also needs to be improved to increase the motivation to learn through e-learning, increasing e-learning readiness. This study strengthens the existing literature in identifying its main antecedents to e-learning readiness during the COVID-19 pandemic. Contextually, namely e-learning in higher education, Indonesia provides information about technology skills and possible challenges and solutions for future policy investments for e-learning readiness towards hybrid learning. Finally, the study helps universities in improving the quality of e-learning learning in the future.

Like most of the previous studies, of course, each has its limitations. This research is limited to the variables of technology skills, equipment capabilities, user satisfaction, and motivation as predictors of student e-learning readiness. Previously, the researcher also found important factors other than these variables, but the limitations and considerations of the research team were determined, so the variables that had been revealed in this study were determined. In addition, this study also has limitations in data collection, especially since the data collection period is quite long, namely from October to December 2021, so there may be differences in the level of e-learning readiness within that period. However, this is done to anticipate the lack of data collected if the charging time is very short.

### Recommendations

Based on the limitations of this study, the researcher recommends further research to reveal various other important factors to increase e-learning readiness, both during the COVID-19 pandemic and not. We also invite further researchers to research and develop methods and results in this study with more varied sample characteristics using more comprehensive measurements. On the other hand, we also provide specific recommendations for universities to analyze the need for using e-learning in terms of student needs. Strengthening digital technology skills and access to equipment is very important for the first time. Then, we expect the lecture service team to provide student satisfaction through optimal service. Finally, we appeal to the entire academic community in higher education to strengthen learning motivation in e-learning.

## Declarations

### Author contribution statement

Wagiran: Conceived and designed the experiments; Analyzed and interpreted the data; Wrote the paper.

Suharjana: Performed the experiments; Contributed reagents, materials, analysis tools or data.

Muhammad Nurtanto: Conceived and designed the experiments; Contributed reagents, materials, analysis tools or data; Wrote the paper.

Farid Mutohhari: Analyzed and interpreted the data; Wrote the paper.

### Funding statement

This research did not receive any specific grant from funding agencies in the public, commercial, or not-for-profit sectors.

### Data availability statement

We have never published and reported the findings in this study. But if general storage is needed, we will consider databased at our university work.

### Declaration of interests statement

The authors declare no conflict of interest.

### Additional information

No additional information is available for this paper.
